# Assessment of heterosis in two *Arabidopsis thaliana* common-reference mapping populations

**DOI:** 10.1371/journal.pone.0205564

**Published:** 2018-10-12

**Authors:** Marieke H. A. van Hulten, Maria-Joāo Paulo, Willem Kruijer, Hetty Blankestijn-De Vries, Brend Kemperman, Frank F. M. Becker, Jiaming Yang, Kathrin Lauss, Maike E. Stam, Fred A. van Eeuwijk, Joost J. B. Keurentjes

**Affiliations:** 1 Laboratory of Genetics, Wageningen University and Research, Wageningen, the Netherlands; 2 Biometris, Wageningen University and Research, Wageningen, The Netherlands; 3 Plant Development & (Epi)Genetics, Faculty of Science, Swammerdam Institute for Life Sciences, Universiteit van Amsterdam, Amsterdam, The Netherlands; Institute of Genetics and Developmental Biology Chinese Academy of Sciences, CHINA

## Abstract

Hybrid vigour, or heterosis, has been of tremendous importance in agriculture for the improvement of both crops and livestock. Notwithstanding large efforts to study the phenomenon of heterosis in the last decades, the identification of common molecular mechanisms underlying hybrid vigour remain rare. Here, we conducted a systematic survey of the degree of heterosis in *Arabidopsis thaliana* hybrids. For this purpose, two overlapping Arabidopsis hybrid populations were generated by crossing a large collection of naturally occurring accessions to two common reference lines. In these Arabidopsis hybrid populations the range of heterosis for several developmental and yield related traits was examined, and the relationship between them was studied. The traits under study were projected leaf area at 17 days after sowing, flowering time, height of the main inflorescence, number of side branches from the main stem or from the rosette base, total seed yield, seed weight, seed size and the estimated number of seeds per plant. Predominantly positive heterosis was observed for leaf area and height of the main inflorescence, whereas mainly negative heterosis was observed for rosette branching. For the other traits both positive and negative heterosis was observed in roughly equal amounts. For flowering time and seed size only low levels of heterosis were detected. In general the observed heterosis levels were highly trait specific. Furthermore, no correlation was observed between heterosis levels and the genetic distance between the parental lines. Since all selected lines were a part of the Arabidopsis genome wide association (GWA) mapping panel, a genetic mapping approach was applied to identify possible regions harbouring genetic factors causal for heterosis, with separate calculations for additive and dominance effects. Our study showed that the genetic mechanisms underlying heterosis were highly trait specific in our hybrid populations and greatly depended on the genetic background, confirming the elusive character of heterosis.

## Introduction

Heterosis, or hybrid vigour, refers to the superiority of F1 hybrids over their “inbred” parents, for any given trait. While the term was coined by Shull in 1914 to replace the word heterozygosis [1] the phenomenon was first described by Koelreuter [2] and later by Mendel and Darwin [3] and has been exploited by humans in agriculture for over a century [4]. Currently, hybrids contribute to crop yield for many species [5], with, for example, more than 95% of corn production relying on hybrid varieties. However, despite the economic relevance of heterosis, the understanding of the underlying molecular mechanisms is still lagging behind. Over the years, three principal genetic models have been proposed to explain heterosis, namely dominance, overdominance and pseudo-overdominance, which have been extensively reviewed elsewhere [6–11]. In brief, the dominance model proposes complementation of various slightly deleterious alleles differentially present in both parents. While the overdominance hypothesis suggests a heterozygous advantage of specific alleles due to novel interactions between the parental alleles at a single heterozygous locus. Lastly, pseudo-overdominance is defined by complementation of slightly deleterious genes tightly linked in repulsion and, therefore, often overlooked as overdominance. In addition to these models, which all relate to cumulative effects of single gene actions, epistasis is expected to play a role in heterosis, referring to novel allelic interactions between different loci in hybrids. While these models have dominated the literature for decades, none of them adequately capture the phenomenon of heterosis completely and recently it has been suggested to abandon their restrictions all together and explain heterosis by simultaneous action of all models mentioned above [8].

In addition to the genetic models described above, increasing evidence for a role of epigenetic regulation in heterosis is emerging [10, 12]. In *Arabidopsis thaliana*, small RNAs, known to mediate gene expression and/or epigenetic regulation, have been observed to be differentially expressed in F1 hybrids compared to both parental lines and their expression was consistent with changes in DNA methylation [13, 14]. Similar observations were made in other species, such as maize [15], and tomato [16]. Although it remains difficult to link these changes directly to heterosis, growth vigour of Arabidopsis F1 hybrids was compromised by reduced DNA methylation [14]. In addition, Lauss et al. [17] recently demonstrated in Arabidopsis that epigenetic divergence is sufficient to cause heterosis.

Heterosis has been studied extensively in allogamous crop species like maize [18], but also in autogamous crops such as cereals [19]. While the self-fertilizing nature of autogamous crops makes the deployability of hybrids in agricultural practice more cumbersome, attempts to generate male sterility–fertility restoration systems for the exploitation of heterosis in such crops have been undertaken [20]. Also in the model plant Arabidopsis, which is a predominantly selfing species, heterosis has been observed for a variety of traits, making this species an excellent choice to elucidate the mechanisms behind heterosis. In Arabidopsis heterosis has been reported for developmental traits such as seedling viability [21], rosette diameter or biomass [17, 22–28], flowering time [17, 22, 29], plant height [17, 23, 30, 31], and seed related traits [22, 27, 32, 33], but also for physiological traits like photosynthetic efficiency [34, 35], phosphate use efficiency [36] and cold tolerance [37, 38]. On the molecular level, nonadditive gene expression has been observed in Arabidopsis hybrids [13, 14, 17] and distinct heterotic metabolite profiles, compared to the parental lines, have been identified [26, 39]. Although most of these studies focused on crosses between two or only a few parental lines, systematic surveys of the degree of heterosis in Arabidopsis indicated extensive natural variation for the potential of hybrid vigour [24, 28]. This opens up the way for genetic mapping approaches in which segregating natural variation might explain differences in hybrid vigour and as such identify genetic factors causal for heterosis. For instance, genome wide association (GWA) mapping of heterotic traits in a large hybrid panel derived from a half diallel mating scheme of 30 different Arabidopsis accessions revealed that nonadditive genetic effects contribute substantially to the genetic variation in this panel [40]. Similarly, genomic analyses of natural variation in rice hybrids identified heterosis-associated superior alleles [41, 42]. In addition, the prevalence of heterosis in maize and its relationship with genetic background and trait characteristics were analysed in a large common reference hybrid population [43].

Here, we examined the range of heterosis for multiple heterotic traits, and the relationship between them, in two largely overlapping Arabidopsis common reference hybrid populations. These populations were generated by crossing a large collection of naturally occurring accessions to two common reference lines. The selected accessions were a subset of a densely genotyped genome wide association mapping panel [44–46], allowing the use of GWA analyses to identify putative candidate genes involved in the regulation of heterosis in these particular populations.

## Material and methods

### Plant material

Seeds of the Arabidopsis HapMap population, collected worldwide and genotyped with 250K SNPs [44–46] were obtained from the ABRC stock centre. Two common reference F_1_ hybrid populations were generated by crossing a subset of the HapMap population to two reference lines, viz. Columbia and Landsberg *erecta*. Ultimately, 100 and 99 hybrids were created with Columbia and Landsberg *erecta* as the common reference parent, respectively (S1 Table). These hybrids, together with their associated parental lines, will hereafter be referred to as the Col-population (population 1) and L*er*-population (population 2). A total of 95 of the Col- and L*er*-hybrids shared a parental line originating from the HapMap panel. To exclude differences due to developmental timing, we selected mainly parental accessions that were early flowering and did not require vernalization for further analysis. However, a few late flowering parental lines were added to estimate the effect of life history differences.

For conveniences, the Col-hybrid population was generated using a specific Columbia line as pollen donor, which contained both GFP and RFP markers under the regulation of the seed-specific Napin promoter [47]. This allowed subsequent identification of true hybrid seeds using fluorescent microscopy as described before [47]. The L*er*-hybrid population was generated using a male sterile Landsberg *erecta* line [48] as recipient parent, consequently resulting in true hybrid seed production. It should be noted that the chosen pollination strategies resulted in a different contribution of possible maternal effects in both hybrid-populations. Whereas the cytoplasm of each hybrid in the second population was derived from the male sterile L*er* line, the hybrid cytoplasms of the first population originated from the different HapMap parental lines. However, this strategy allowed for the generation of hybrid seeds by hand-pollination of unopened flower buds without emasculation, leaving several axillary flower meristems intact. HapMap parental lines and common reference lines were maintained by selfing. No significant differences in phenotypic values were observed when comparing manual pollination versus selfing (S2 Table), likely because emasculation was not required for the production of hybrid seeds and several axillary flower meristems could be left intact. Plants used for maintaining parental lines and for generating the associated F1 hybrid seeds were grown simultaneously under the same conditions. Moreover, for the Col population hybrid seeds and fresh parental seeds were collected from the same parental plant.

### Plant cultivation

Seeds were stratified for at least four days at 4°C. After imbibition seeds were sown on 4x4cm rockwool blocks (MM40/40, Grodan B.V.) pre-soaked in 1 g.L^-1^ HYPONeX fertilizer (nitrogen:phosphorus:potassium, 7:6:19; Hyponex, Osaka, Japan). Ten replicates per genotype were grown in a randomized block design in a controlled climate chamber (20/18°C day/night) with 16 hours of light (125 μmol photons m^-2^.s^-1^) at a relative humidity of 70%. Plants from population 1 received 1 g.L^-1^ fertilizer solution (HYPONeX) by automatic flooding a flow table for five minutes three times a week, with additional watering when needed during the flowering stage. Plants from population 2 received fertilizer daily in the first week, followed by two times in the second week and three times a week thereafter. During the flowering stage, plants from population 2 were watered manually with 1 g.L^-1^ fertilizer solution (HYPONeX) once a week by standing water, supplemented with tap water when needed. For growing of each parental-hybrid combination we strived to use seeds that were propagated simultaneously to reduce differences in maternal environmental conditions.

### Collection of phenotypic data

All surviving plants were phenotyped for leaf area, flowering time, total plant height, main stem branching, rosette branching, seed yield, seed size, 1,000 seed weight and number of seeds per plant. Leaf area (LA) was measured using an in-house developed automatic imaging system (Open Pheno System). This system captured plant growth by taking pictures from above at set time points. These pictures were processed in ImageJ to analyse projected leaf area. Images were captured on an hourly basis until approximately 17 days after sowing. After this time calculated leaf areas were less reliable due to partly overlap of individual plants. Flowering time was scored in days after sowing upon opening of the first flower. After initiation of flowering, the Col-hybrid population and its parental lines (population 1) were left in the climate chamber, whereas the L*er*-hybrid population and its parental lines (population 2) were transferred to controlled greenhouse conditions. After transfer, plants were no longer kept at random but instead the replicates of the HapMap accession and associated hybrid were grouped together. For population 1, individual replicates were pooled for seed yield measurements due to lack of manpower. However, for population 2, replicates were measured individually. Only heterozygous L*er Msms* plants, displaying wild type phenotypes, were analysed for inflorescence architecture traits and seed traits to reduce the impact of plant sterility on those traits. Inflorescences were kept upright throughout the experiment and left to grow until the end of flowering. Plants were cut just underneath the rosette and placed in paper bags for further ripening. As the timing of cutting was decisive for seed related traits, in general, inflorescences were placed in bags when the last siliques had filled up and had just started to show the first signs of yellowing. Cutting to soon resulted in insufficient maturation of (some of) the seeds and might hence influence seed yield traits, while cutting to late resulted in seed loss due to pod shattering. After complete ripening inflorescence architecture was measured and seeds were collected. Total plant height (HT) was measured in cm. from the base of the inflorescence until the last silique on the main stem. Main stem branching (MSB) was recorded as the number of side branches on the main stem, while rosette branching (RB) was recorded as the number of side branches originating from the base of the rosette. Seed yield (SY) was determined by weighing pooled seed batches (for population 1) or individual seed batches (for population 2) and calculated as grams of seeds produced per plant. Seed size (SZ) was determined for subsamples of the seedbatches of approx. 100 seeds using a tool incorporated in GERMINATOR, a software program originally designed for automatic scoring and evaluation of germination [49]. One of the features of GERMINATOR is that it can automatically detect individual Arabidopsis seeds from an image and measures the length and width of the seed. The subsample was subsequently weighted and 1,000 seed weight (SW) was calculated. The number of seeds per plant was estimated by dividing SY by individual SW.

### Statistical analysis

Mid parent heterosis **(**MPH) levels were calculated as: MPH = (mean F_1_ –mean P_MPV_)/mean P_MPV_ in %, where P_MPV_ represents mid parent values, the average of both parental lines. BPH was calculated as: BPH = (mean F_1_ –mean P_best_)/mean P_best_ in %, where P_best_ represent the best performing parental line (Falconer and Mackay, 1996). For the GWAs analysis, absolute mid parent heterosis (MPH_ABS_) levels were calculated as: MPH_ABS_ = mean F_1_-mean P_MPV_)

Correlations between traits were determined by calculating Spearman’s rho (*r*_*s*_) correlation coefficients in version 24 of IBM SPSS Statistics using a two-tailed significance test. R^2^ values represent squared Pearson correlation coefficients (*r*) calculated in SPSS using a two-tailed significance test. Absolute trait values, e.g. when comparing HT of a hybrid line with HT of its best parental line, were compared using a paired-samples *t*-test. The coefficient of variation (CV_G_) was calculated as CV_G_ = √(V_G_)/ $\bar{x}$ *100%, where V_G_ is the genetic variation calculated as the variance among genotypes and $\bar{x}$ is the population mean. Broad-sense heritability was calculated using the formula H^2^ = V_G_/(V_G_+V_E_)_,_ where V_E_ represents the environmental variation, calculated as the mean of the sum of squares within genotypes.

Correlations between genetic distance (GD) between the parental lines and heterosis levels of the respective hybrids were evaluated by calculating Spearman’s *r*_*s*_ in SPSS using a two-tailed significance test. As a proxy for genetic distance between the parental lines of all hybrids, we used a kinship matrix for the Arabidopsis HapMap population, build to correct for population structure in genome wide association studies [50]. Instead of the fluorescent-tagged Col line [47] and the male sterile L*er* line [48], kinship data for Col-0 and L*er*-1 was used, respectively.

GWA mapping was conducted in R using a mixed model approximation incorporated in Scan-GLS [50] and the publicly available 250k Arabidopsis SNP data [44]. Dominant effects (d) were estimated by mapping MPH_ABS_ using a dominant SNP encoding model of 1 for heterozygous SNPs and 0 for homozygous SNPs, relative to the reference parental allele (Col-0 or L*er*-1). Additive effects (2a) were estimated by mapping the mean of the parental lines using an additive SNP encoding model of 0 for the presence of two times the non-reference parental allele and 1 for the presence of two times the reference parent (Col-0 or L*er*-1) allele. SNPs with a MAF < 0.05 were omitted from analysis. For the analysis of seed traits data of accession Ha-0 (CS28336), and derivatives thereof, were removed prior to GWA mapping. This accession, and its derived hybrids, yielded substantially larger seeds compared to all other accessions studied here, which confounded mapping of those traits.

## Results

### Development of two Arabidopsis common reference hybrid populations

To perform a systematic study of the extent of heterosis for multiple traits in Arabidopsis, two largely overlapping common reference hybrid populations were generated. For this purpose a subset of the Arabidopsis HapMap population [51] was crossed to two common laboratory reference accessions in Arabidopsis-research, namely Columbia (Col) and Landsberg *erecta* (L*er*). Ultimately, 100 and 99 hybrids were created with Columbia and Landsberg *erecta* as the common reference parent, respectively (S1 Table). These hybrids, together with their associated parental lines, will hereafter be referred to as the Col-population (population 1) and L*er*-population (population 2). A total of 95 of the Col- and L*er*-hybrids shared a parental line originating from the HapMap panel.

### Quantitative variation for developmental and yield related traits

For both hybrid populations nine traits were analysed, simultaneously with the associated parental lines in two independent experiments. The first experiment encompassed the Col-hybrid population and its associated parental lines while the second experiment analysed the L*er*-hybrid population and its associated parental lines. The developmental and yield related traits under study consisted of: projected leaf area (LA) at 17 days after sowing (DAS), flowering time (FT), entailing DAS at opening of the first flower bud, height of the main inflorescence (HT), number of side branches originating from the main stem (MSB) or from the rosette base (RB), total seed yield (SY) in grams of seeds produced per plant, 1,000 seed weight (SW), seed size (SZ) and an estimation of the number of seeds produced per plant (NS). For most traits substantial variation was detected among the parental lines and among the hybrids (Table 1), indicative of heritable genetic variation for the traits under study. Broad sense heritability estimates (H^2^) were calculated by estimating the proportion of the phenotypic variance that is attributable to genetic factors (Table 1). Interestingly, broad-sense heritabilities of the hybrids were in most cases lower than those of the parental lines (*p* = 0.027, two-tailed paired t-test). However, this coincided in general with lower coefficients of genetic variation observed for the hybrids, implying that the lower broad sense heritability estimates observed in the hybrids was due to an overall lower degree of phenotypic variation between them (Table 1, S1 Fig). This suggests that hybridization of two inbred lines has a buffering effect on the phenotypic space of most traits analysed, as can be judged from the narrower distributions of trait values (S1 Fig). It must be noted, however, that the hybrids share the genome of the common parent and are thus more closely related to each other than the parental lines.

**Table 1:** Descriptive statistics for diverse traits in population 1 and 2. Population 1 represents the Col-hybrid population including its associated parental lines, whereas population 2 represents the L*er*-hybrid population including its associated parental lines. Abbreviations used: AVG, population average; SD, standard deviation; LA, projected leaf area in cm^2^ 17 days after sowing; FT, flowering time in days after sowing of opening of first flower; HT, height of the inflorescence in cm; MSB, main stem branching in number of branches originated on the main stem; RB, rosette branching in number of branches originated from the base of the rosette; SY, seed yield in grams of seeds produced per plant; SW, 1000 seed weight in estimated grams of 1000 seeds; SZ, seed size in μm^2^*10^3^; NS, an estimation of the number of seeds produced per plant; H^2^, broad-sense heritability; CV_G_, coefficient of genetic variation; *r*_*s*_, correlation between expected mid parent values and the actual values as measured in their offspring (Spearman), with associated p-values (*p*); n.d., not determined.

		Parental lines				Hybrids					
Trait	population	AVG	SD	H^2^	CV	AVG	SD	H^2^	CV	*r*_*s*_	*p*
LA	1	4.53	1.00	0.27	22.16	5.81	1.34	0.28	23.00	0.04	0.68
	2	2.28	0.49	0.27	21.58	2.87	0.57	0.17	19.81	0.28	0.01
FT	1	29.35	4.81	0.67	16.37	28.87	5.30	0.84	18.36	0.64	0.00
	2	32.17	4.96	0.81	15.40	28.32	3.75	0.75	13.23	0.71	0.00
HT	1	49.73	8.51	0.80	16.98	52.20	5.74	0.51	11.00	0.55	0.00
	2	46.76	6.80	0.65	14.53	54.41	5.11	0.55	9.38	0.66	0.00
MSB	1	5.50	1.61	0.76	29.33	5.39	1.25	0.63	23.20	0.45	0.00
	2	5.38	1.77	0.60	32.89	4.92	1.15	0.41	23.25	0.72	0.00
RB	1	3.63	1.17	0.48	32.20	2.52	1.13	0.44	44.71	0.17	0.12
	2	2.75	1.40	0.60	50.71	2.15	0.87	0.46	40.58	0.43	0.00
SY	1	0.27	0.10	n.d.	37.90	0.32	0.11	n.d.	33.80	0.70	0.00
	2	0.18	0.07	0.51	38.23	0.20	0.06	0.38	30.15	0.48	0.00
SW	1	25.30	2.25	n.d.	8.91	26.15	1.88	n.d.	7.19	0.56	0.00
	2	29.50	9.56	n.d.	32.41	34.36	7.56	n.d.	22.02	0.14	0.21
SZ	1	82.38	7.99	n.d.	9.70	81.68	6.45	n.d.	7.90	0.58	0.00
	2	66.43	7.99	n.d.	12.03	70.08	4.55	n.d.	6.49	0.42	0.00
NS	1	10827.62	3943.86	n.d.	36.42	12326.79	3970.75	n.d.	32.21	0.69	0.00
	2	6368.82	2962.38	n.d.	46.51	6066.29	2111.83	n.d.	34.81	0.50	0.00

**Fig 1:**
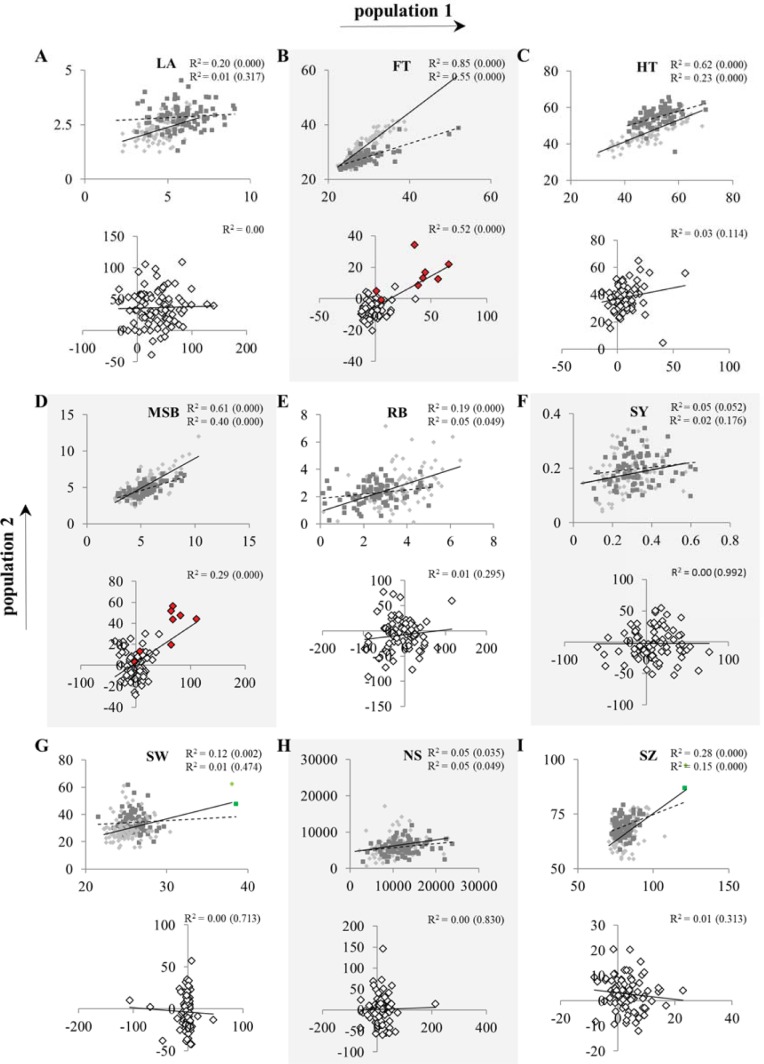
Comparisons across populations for different traits. Shown are LA (A), FT (B), HT (C), MSB (D), RB (E), SY (D), SW (G), NS (H), and SZ (I). In each graph data of the Col-population (population 1) is plotted on the x-axe and data of the L*er*-population (population 2) is plotted on the y-axe. Trait values of parental lines (light grey diamonds) and hybrid lines (dark grey squares) are shown in the top graphs, while the bottom graphs contain information on mid parent heterosis (MPH) values of the hybrids (open diamonds). Axes units of the top graphs are in cm^2^ for LA, days after sowing for FT, cm for HT, absolute numbers for MSB and RB, grams of seeds per plant for SY, mg per 1000 seeds for SW, absolute numbers for NS, μm^2^*10^3^ for SZ, and percentage of heterosis for MPH. Axes of the bottom graphs represent percentages of MPH levels. Trendlines are represented by solid lines for trait values of parental lines or MPH levels and dotted lines for trait values of hybrid lines. Squared Pearson correlation values (R^2^) are shown in the right corner of each graph for parental lines (top) and hybrid lines (bottom), with the associated *p*-values indicated between brackets. Open diamonds highlighted in red in the MPH values graph for FT and MSB represent hybrids heterozygous at a specific locus on chromosome 4 that was highly associated with heterosis for both traits. Diamond and square highlighted in green in SW and SZ graph represent parental line Ha-0 and its associated hybrids. Abbreviations are as listed in the legend of Table 1.

To determine the effect of possible environmental variation and genetic background of the hybrids we compared the data of the two populations grown in different experiments. Because 95 of the parental lines were grown in both experiments this allowed us to determine the effect of experimental variation on isogenic lines, whereas the hybrids shared the contribution of the 95 accessions but differed in the genetic counterpart of hybridization (Col vs. L*er*). As expected, comparing the same traits resulted in lower correlations between the hybrid lines of the two populations than were observed between the parental lines of the two populations (Fig 1). However, even for the parental plants the similarity between the two experiments varied tremendously for some traits. Strong correlation between data of the two experiments for parental lines that were identical in the two population was observed for FT, HT, and MSB (Fig 1B, 1C and 1D), while much weaker correlation was observed for LA at 17 DAS, RB, and all seed-related traits. Although both experiments were initially performed in the same climate chamber under identical growing conditions, slight experimental variation, *e*.*g*. in watering regimes, is illustrated by the on average larger plants at 17 DAS in population 1 compared to population 2. In addition, seed-related trait differences between the two experiments were observed. On average higher seed yields were obtained in population 1 than in population 2. Moreover, SZ was slightly increased in population 1. Finally, higher variation for SW was detected in population 2 (Table 1; Fig 1). These seed related effects might be caused by the transfer of population 2 to less controlled greenhouse-conditions after the transition to flowering, while population 1 was kept at highly controlled conditions in the climate chamber until the end of the experiment. Likely, environmental differences, predominantly in light quality and temperature, during seed maturation, possibly in combination with the observed differences in plant size at 17 DAS, have influenced the seed-related traits. Taken together, these results indicate a higher GxE interaction for some of the traits studied here. For those traits that appeared less robust under the tested conditions further between-population comparisons should be considered with caution.

### Occurrence of Heterosis for multiple traits in Arabidopsis

To assess the prevalence and extent of heterosis in the two common reference hybrid populations, Mid Parent Heterosis (MPH) levels for each of the traits were determined for all the hybrids. MPH is a relative measure of the degree by which the hybrids exceed expectations based on the average performance of both parents, in contrast to Better Parent Heterosis (BPH), where trait values of hybrids are compared only to the trait value of the best performing parent. While BPH may be considered economically more relevant than MPH, since a high BPH guarantees that the offspring exceeds the maximum capacity of the parental lines, we focused primarily on MPH values. MPH is less sensitive to extreme parental trait values and assures that both parents are considered in determining the level of heterosis. This allowed us to directly compare hybrids within each population since these shared a common reference parent. Consequently, the differences in MPH values could be fully attributed to differences between the HapMap parental accession of the hybrid, regardless whether this parent was the best performing one or not.

**Fig 2:**
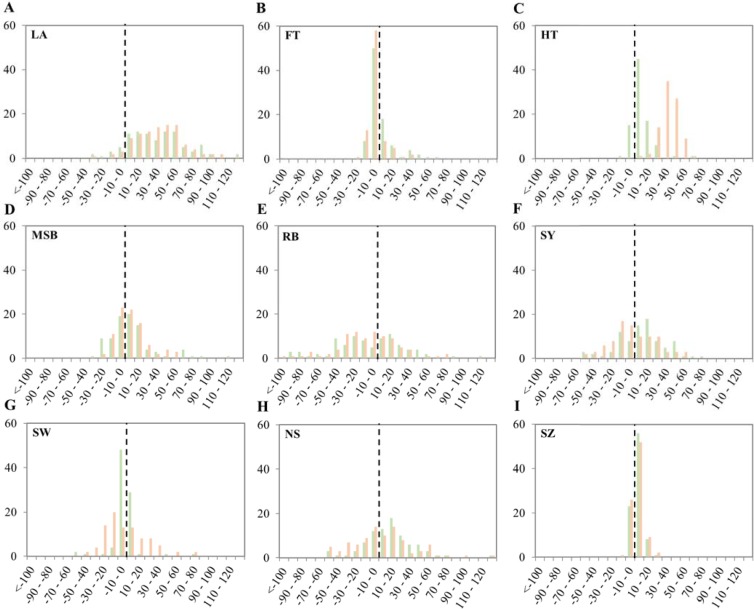
Distribution of mid parent heterosis for diverse traits in both hybrid populations. Shown are frequency distributions of mid parent heterosis for LA (A), FT (B), HT (C), MSB (D), RB (E), SY (E), SW (G), NS (H), and SZ (I). Green bars represent Col hybrid population. Red bars represent L*er* hybrid population. Abbreviations are as listed in the legend of Table 1.

Substantial variation in the level of heterosis could be observed between different hybrids although the extent of heterosis was highly trait-dependent (Fig 2). Predominantly positive heterosis was observed for LA at 17 DAS, and HT, while mainly negative heterosis was observed for RB. For most other traits both positive and negative heterosis was observed in roughly equal amounts. For FT and SZ, however, only low levels of heterosis were detected, with most hybrids showing a MPH of less than (-)20%. For SZ this coincided with low coefficients of variation for absolute trait values (Table 1). This suggests that SZ is relatively well conserved between different Arabidopsis accessions, resulting in a possible constraint on phenotypic variation for this trait. That said, incidental observations of accessions with exceptional large seed size have been reported as well [32]. The narrow distribution of heterosis observed for FT may reflect the restriction that was applied on the genetic background of the hybrids, since the parental lines were selected to be predominantly early flowering. Nonetheless, some of the hybrids flowered substantially later than their parents.

While the degree of MPH was highly trait-dependent, for most traits heterotic ranges were remarkably similar between both hybrid populations, reflecting the strong correlation observed between experiments for some traits (see also below). The only exceptions to this high coincidence of MPH distributions were HT and SW (Fig 2). For the latter trait, the range of distribution in the L*er*-hybrid population was wider in both directions. This coincided with the more extensive variation that was observed for this trait in population 2 compared to population 1 (Table 1) and might be the result of the different environmental conditions during seed maturation. In addition, L*er*-hybrids showed on average 38% MPH for HT, whereas Col-hybrids showed on average only 7% MPH for this trait. This indicates that for HT the common reference parents have a differential effect on the level of MPH. The common reference parent of the L*er*-hybrid population is known to carry a mutation in the *Erecta* locus, which has been associated, among others, with a reduced inflorescence height (reviewed by [52]), which ultimately results in lower expected mid-parent values (MPV) for L*er*-hybrids. However, BPH levels in this population were also predominantly positive (S2 Fig), suggesting a high degree of (pseudo-)overdominance. In total 64 of the 99 analysed L*er*-hybrids displayed significantly longer inflorescences than their best parent, in most cases being the HapMap accession (*p*<0.05). This indicates that the higher MPH levels for HT in the L*er*-hybrid population are not solely the result of complementation of the *erecta* mutation in the common reference parent, but that a different genetic model must underlie the heterosis mechanism for this trait in this specific population.

For most traits no correlation could be detected between MPH values of the two hybrid populations, with the exception of FT and MSB, which showed a high and moderate correlation, respectively. This suggests relative simplicity in the regulation of FT and MSB and genetic similarity between the two common reference parents at the loci involved in the regulation.

### Heterosis for projected leaf area established during early development

The most extensive and predominantly positive heterosis in both hybrid populations was observed for LA at 17 DAS, although the absolute trait values were highly GxE dependent (Fig 2A). Nonetheless, this trait was studied in more detail in population 2 by daily measurement of LA using an in-house developed automatic imaging system [53]. Visual inspection of growth curves indicated a clear separation of all hybrid genotypes from the poorest performing parental lines from very early timepoints onwards (S3 Fig).

Hybrid seedlings were on average earlier (2.5–5 DAS) detected by the imaging system than parental lines (S4 Fig). Given the resolution of the camera system this variation may represent differences in germination rate or seedling establishment. However, before the beginning of the experiment seeds were stratified to break possible dormancy. In addition, almost all seeds were obtained from simultaneous propagation and generation of parental and hybrid lines, respectively, ruling out differences in maternal environmental conditions. Therefore, the observed differences in germination rate and/or seedling establishment between hybrid and parental lines can be considered as a heterotic trait as well.

To exclude differences in germination rate and/or seedling establishment between hybrids and parental lines for growth analyses, all growth curves were normalised. For each individual plant, t_0_ was assigned to the day that the projected leaf area became larger than 2 mm^2^, corresponding to growth stage 0.7–1.0, where cotyledons emerge and start to expand [54]. Although the extent of heterosis was reduced after this correction, predominantly positive heterosis levels could still be observed for LA at all timepoints after t_0_ (S5A Fig). Interestingly, MPH levels increased only marginally in time, peaking in most hybrids at 8 days after t_0_ (S5B Fig). This suggests that heterosis for LA is already determined at relatively early growth stages and is maintained at similar levels throughout the growing period.

### Correlation between heterosis levels for different traits follows trait correlations

**Fig 3:**
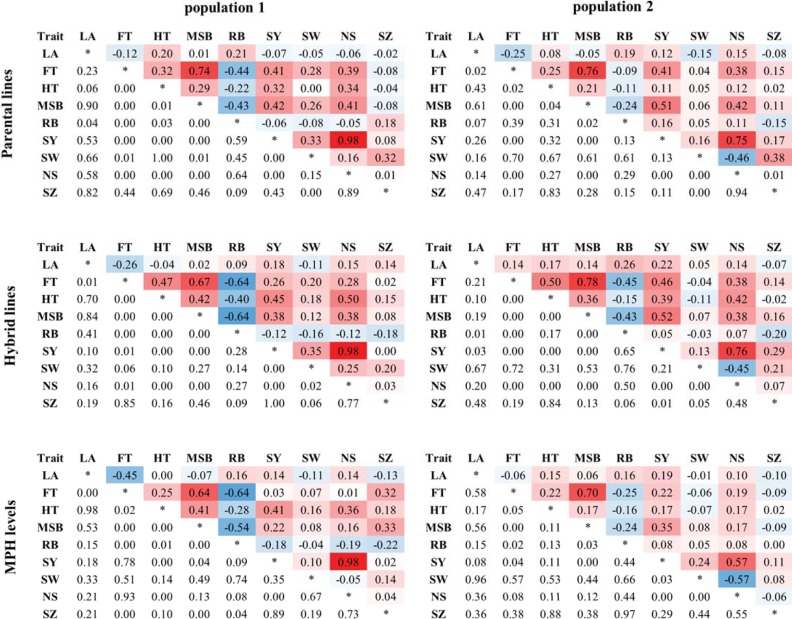
Spearman’s rank correlations of trait values for parental lines, hybrids and MPH levels in both populations. Shown are correlations (r_s_) between all analysed traits in the parental lines of population 1 (Col population) and 2 (L*er* population), the hybrids of population 1 and 2 and the MPH values of the hybrids of population 1 and 2 (top right) and the corresponding *P*-values (bottom left). Different colours represent the strength and direction of the correlation with red representing a positive correlation and blue a negative correlation. Abbreviations are as listed in legend of Table 1.

To unveil trait relationships, all measured traits were subjected to Spearman rank correlation analysis for parental lines, hybrids, and MPH values separately (Fig 3). Taken together, for the traits MSB, FT, SY, HT, and NS, a variety of correlations with other traits were found, while projected LA at 17 DAS, SW, and SZ showed hardly any correlation with any of the other traits under study. A remarkably high correlation was detected in both populations between SY and NS, not only between the parental lines of the two experiments, but also between the two hybrid-populations and even between MPH values of the two hybrid-populations. Interestingly, no correlation was found for SY and SW in any combination tested, indicating that in our experimental set-up total seed yield in Arabidopis seems mostly determined by the number of seeds produced by a plant and not by the weight of individual seeds. Another strong positive correlation was observed between FT and MSB. In addition to MSB, FT showed weaker but significant correlations with RB and HT in almost all combinations tested (Fig 3).

In general, correlations between MPH values of any given two traits followed largely the same trends as those observed between the trait values in the parental lines, indicating that heterosis is highly trait or trait-family specific. This suggests that, at least in Arabidopsis, heterosis is not a whole-genome phenomenon that affects all traits equally.

### Heterosis is independent of genetic distance between parental lines

Being able to predict hybrid performance remains of tremendous interest in agriculture. Therefore, we analysed to which extent the performance of the hybrids could be anticipated based on the performance of their parental lines. However, the ability to predict this varied greatly among traits, illustrated by correlation coefficients between hybrid performance and mid parent values ranging from *r*_*s*_ = 0.04 for LA at 17 DAS in the Col-hybrid population to *r*_*s*_ = 0.72 for MSB in the Col-hybrid population (Table 1). Rationally, the correlation between hybrid and parental performance decreases with increasing heterosis levels, since hybrid performance will deviate farther from parental performance with increasing heterosis levels. Here, we investigated whether heterosis levels could be explained by estimating the genetic distance between the parental lines. It is expected that the chance for new, possibly favourable, interactions between parental alleles to arise increases when there are simply more polymorphic alleles. For this reason the relationship between heterosis and parental genetic distance was analysed. As a proxy for genetic distance between the parental lines of each individual hybrid a kinship matrix for the Arabidopsis HapMap population was constructed, which was originally designed to correct for population structure in genome wide association studies [50]. In both populations hardly any relationship could be detected between heterosis levels in the hybrids and genetic distance between the parental lines that gave rise to the hybrids (S3 Table). This implies that other mechanisms than genetic distance affects heterosis in the set of Arabidopsis hybrids that was tested here.

### Genome wide association mapping of heterotic traits

**Fig 4:**
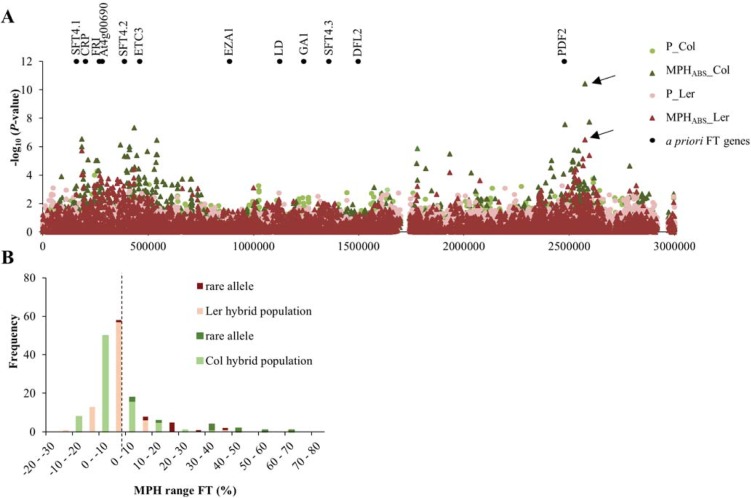
Genome wide association mapping of heterosis for flowering time. Shown are local distributions of the–log_10_(*P*-value) of SNPs associated with MPH_ABS_ or parental means for FT in population 1 (Col) and population 2 (L*er*), relative to the location of *a priori* genes involved in FT at the beginning of chromosome 4. Black arrows indicate the position of SNP m129800 (A). The allele frequency distribution of SNP m129800 over different relative MPH classes for FT are given in (B) with green representing MPH classes in population 1 (Col) and red representing population 2 (L*er*). The distribution of hybrids that were heterozygous for SNP m129800, by carrying the rare allele in addition to the Col/L*er* allele of the common reference, are indicated in dark green or dark red, while the hybrids that were homozygous for this SNP are indicated in light green or light red. Abbreviations: FT, flowering time; MPH_ABS_, absolute mid parent heterosis levels; MPH, relative mid parent heterosis levels.

To analyse the genetic constitution of heterosis for the traits investigated here, GWA mapping was performed. For this, the publicly available 250k single nucleotide popymorphism (SNP) data of the accessions of the Arabidopsis HapMap panel [44–46] and a mixed model approximation incorporated in Scan-GLS [50] was used. For each trait absolute heterosis values (MPH_ABS_) were analysed in contrast to the relative MPH levels that had been used for the phenotypic description of the two populations. The rationale behind this was that additive effects are not expected to contribute to MPH_ABS_ since MPH_ABS_ is defined by the performance of the hybrids minus the average performance of both parents (P_MPV_), which will cancel out additive effects. As a consequence, mapping MPH_ABS_ will allow us to identify predominantly dominant acting SNPs. Dominant effects (d) were estimated by mapping MPH_ABS_ values using a dominant SNP encoding model of 1 for heterozygous SNPs and 0 for homozygous SNPs. *P*-value correction according to Benjamini and Hochberg’s FDR-controlling procedure for multiple testing [55], yielded significant associations (p<0.05) for only 8 out of the 36 studied traits (S4 Table). Since only a limited number of positive associations were found it was decided to apply an arbitrary threshold of -log_10_(*P*-value)>4 on the uncorrected P-values to reduce the number of false negatives. This method has been demonstrated previously to result in enrichment of *a priori* candidate genes [44]. This way, despite the relatively small population size of less than a hundred genotypes, between 10 and 218 SNPs were detected that were significantly associated with heterosis (S4 Table, S5 Table, S6 Fig). It should be noted however, that more false positives may have been generated by this method. To include SNPs that may have a more additive character for the traits under study, additive effects (2a) were estimated by mapping the mean of the parental lines using an additive SNP encoding model of 1 for the presence of two times the non-reference parental allele and 0 for the presence of two times the reference parent (Col-0 or L*er-1*) allele. This approach identified between 7 and 52 significant SNPs (-log_10_(*P*-value)>4; S4 Table, S5 Table). There was little to no overlap between the sets of significant SNPs identified for MPH_ABS_ with a dominant encoding SNP model and parental means with an additive encoding SNP model. For this reason the ratio of dominance (d/a) could not be adequately calculated. For most traits only few associations were detected, which were relatively weak. In general more SNPs were identified after mapping MPH_ABS_ using a dominant encoding model than mapping parental means using an additive encoding model, with an exception for seed related traits (S4 Table). In total 1,218 SNPs were identified that were significantly associated (-log_10_(*P*-value)>4) with variation for at least one of the studied parental trait values or with variation for MPH_ABS_ levels for these traits. Most of the significantly associated SNPs were uniquely detected in only one of the traits under study and in only one of the populations, even although up to 94% of the parental accessions were represented in both populations (S5 Table). However, there was some similarity detected between significant SNPs for MPH_ABS_ levels of FT, MSB and RB. For instance, the SNP with the highest -log_10_(*P*-value), namely m129800 located on chromosome 4, was found to be significantly associated with MPH_ABS_ for FT and MBS in both populations and MPH_ABS_ for RB in the Col-population (S5 Table). All significant associations within this region were specific for heterosis, since these SNPs were not found to be significantly associated with the parental FT and MSB values (Fig 4A, S5 Table, S6 Fig). Chromosome four of Arabidopsis is known to harbour several flowering time QTLs and *a priori* candidate genes, including the *FRIGIDA* (*FRI*) gene. [45, 56–58]. Although SNPs within the *FRI* gene itself were not significantly associated with FT or MSB in our analysis, some displayed elevated -log_10_(*P*-value) scores. SNP m129800 itself was located 100 Kbp downstream of the *a priori* gene *PDF2* (Fig 4A). Although m129800 was not in LD with *PDF2*, as determined with the LD tool in the GWA-portal [59]. The rare (non Col/L*er*) allele variant of this most significant SNP was present in 9 and 10 parental accessions in population 1 and 2, respectively. Heterozygosity at this locus was predominantly associated with high relative MPH values for flowering time (Fig 4B). Moreover, hybrids heterozygous at this locus (highlighted in red in Fig 1B and 1D) determined for a large part the correlation between MPH for FT and MSB.

Here we explored the possibility to use genome wide association approaches to map heterosis. Although the feasibility is largely depending on the GxE interactions of the phenotype under study, promising regions that may contain causal genes for heterosis for specific traits were identified in this study. However, further study will remain essential to confirm the identified associations and elucidate the possible role of the underlying candidate genes in heterosis.

## Discussion

### Heterosis for multiple traits

A wide range of heterotic responses was observed in the large set of Arabidopsis hybrids that was studied here, all sharing either Col or L*er* as a common parent. However, the extent of heterosis was very trait specific. Heterosis was prevalent for LA at 17 DAS, even after correction for germination and/or seedling establishment. In contrast, on average less heterosis was observed for all other traits. Previously, extensive Better Parent Heterosis was observed for nearly every trait under study in maize [43]. Nevertheless, the extent of heterosis in the autogamous species Arabidopsis observed here is in line with the generally much smaller amount of heterosis in autogamous than in allogamous crops [60]. Possibly, purifying selection of slightly deleterious alleles is stronger in autogamous plants resulting in mainly overdominant and epistatic mechanisms contributing to heterosis. Reducing the contribution of dominant mechanisms might ultimately lower the total amount of heterosis observed. Moreover, the genetic architecture of an outcrossing crop like Maize has resulted in the occurrence of regions were genes are inherited as a single block due to a low recombination frequency, *e*.*g*. around centromeres (reviewed by [61]). The manifestation of such haplotype blocks can be the origin of heterosis via the pseudo-overdominance principle, in which different haplotype blocks are combined in hybrids of diverse Maize inbred lines. In contrast, in self-pollinating species like rice and wheat, epistasis is thought to play a bigger role in heterosis [61, 62]. The latter might also be the case in Arabidopsis, especially for the polygenic traits under study here.

In our set-up, the extent of heterosis was highly trait-dependent, with most heterosis observed for LA at 17 DAS in both hybrid populations. Similar results have previously been obtained in Arabidopsis and maize, respectively [22, 43]. In line with our findings, high mean MPH values for rosette diameter and plant biomass, traits related to LA, were reported for five Arabidopsis hybrids. Interestingly, mean MPH values for flowering time, seed yield, 1,000 seed weight and the number of seeds per plant were much lower in the five hybrids studied [22], in agreement with the extent of MPH that was observed for similar traits in our Col- and L*er*-hybrid populations. It is tempting to speculate that trait-dependency of the extent of heterosis is related to the extent of phenotypic variation among the parental lines observed population wide, with higher heterosis levels for traits that are more diverse within the population.

### LA and early development

On average, in population 2, hybrid seedlings were detected by our imaging system sooner than parental lines (S4 Fig). These observed differences in germination rate and/or seedling establishment between hybrid and parental lines may be considered as a heterotic trait in itself. However, in our experimental set-up the effect of pollination strategy on F_1_ hybrid seed size and weight and hence on germination, seedling vigour and plant establishment cannot be completely ruled out since F_1_ hybrid seed size and weight were not measured. Although in our study no big differences in manual pollination versus selfing were observed (S2 Table), different pollination strategies have previously been shown to have an effect on F_1_ seed size and subsequent shoot biomass [24]. However, in the study of Meyer et al., hybrid seed production was restricted to five to six flowers per plant and similar size advantages were accomplished when seed harvesting of purely selfing plants was equally restricted, indicating a strong influence of positional resource allocation on the developing seeds. In our study, hybrids were generated by hand-pollination of unopened flower buds without emasculation, leaving axillary flower meristems intact. Using a similar pollination method, no significant differences on F_1_ hybrid seed size or subsequent biomass production was detected previously [27].

After correction for germination rate and seedling establishment, MPH levels for LA increased only minimally over time, suggesting that heterosis for this trait originates at early time points in Arabidopsis and is sustained thereafter. This is in line with previous results in Arabidopsis where heterosis for similar traits was detected as early as 4 to 10 DAS [24–26]. Moreover, an enhanced metabolic activity during early development in hybrids was reported, with most changes occurring between 4 and 6 DAS [26].

### Similarities and discrepancies between two hybrid populations

High MPH values for both FT and MSB were found in the subset of Col- and L*er*-hybrids that shared the same HapMap parental accessions. This suggests that the mechanisms underlying heterosis for those traits in these particular hybrids may be shared, and is indifferent of a Col or L*er* genetic background. Indeed, it is known that Col and L*er* are highly similar in the regulation of flowering time, which is mainly controlled by a few strong-effect loci [63], that also affect architectural traits like branching[64].

Interestingly, no correlation of heterosis for HT was detected between the two hybrid populations, while the correlation of parental lines between the two experiments was much stronger for this trait. This indicates that heterosis for HT is strongly influenced by the Col or L*er* genetic background. Both MPH and BPH levels were higher for this trait in the L*er*-hybrid population (S2 Fig). Likely, this is caused by the mutated and non-functional *erecta* locus harboured by the common parent of the L*er*-hybrid population, which has been associated before with heterosis for length of the main stem, number of buds, flowers, and fruits, fresh weight, and dry weight caused by overdominanance when present in a heterozygous state with a wildtype locus [30]. SNPs in or nearby the *Erecta* locus were not found to be significantly associated with heterosis for HT in our study. This further indicates that the heterosis observed in the L*er* hybrids is determined by the common reference parent and much less by variation in the *Erecta* locus of the HapMap parents. While the presence of a heterozygous *Erecta* locus in the L*er*-hybrid population may very well have enhanced heterosis for HT in this population, the extent of heterosis for this trait in the L*er*-hybrid population is likely caused by additional loci. However, complementation of rare recessive alleles present in either common reference line, such as the extreme case of the *erecta* allele (n = 1), are not likely picked up by GWA.

### Correlations between MPH levels follow trait correlations

If heterosis would be caused by a single mechanism one would expect heterosis for different traits to be correlated. However, heterosis appeared to be highly trait or trait-family specific (Fig 3). In general, significant correlations identified between MPH levels for different traits were often also observed between these traits in the parental lines. For instance, MSB and FT were related, both within the parental lines, within the hybrids and when comparing MPH levels for these traits in both hybrid populations (Fig 3). Likely, traits with significant correlations share a common genetic and/or physiological basis. Higher MSB may be the result of genotypic differences in developmental timing. Previously, opposite effects have been reported for the correlation between flowering time and main stem branching. A negative correlation was reported by [65], while positive correlations were reported by [66] and more recently by [67]. The results described here could be due to the selection of predominantly early flowering accessions in our set-up. Together with the results of [65],[66], and [67] they may suggest that the correlation between flowering time and main stem branching is not linear. A reduced stem branching due to absence of elongation of cauline axillary meristems has been observed in some late flowering lines, and several of the identified QTLs for this trait were found to co-localize with known flowering time genes [64]. However, in early-flowering genotypes fewer axillary meristems were detected. While both a reduced stem branching and the development of fewer axillary meristems negatively affect the total number of elongated axillary meristems, they were associated with opposite flowering time phenotypes, supporting a non-linear correlation between flowering time and main stem branching. In contrast to MSB, the correlation between RB and FT was inversed. In general, less side branches from the base of the inflorescence were observed in our experimental set-up when plants flowered slightly later. Besides MSB and RB, FT also correlated moderately with HT. A partial correlation between flowering time and height in Arabidopsis accessions has been described previously [66, 68]. In addition, it has been reported that genes involved in the regulation of FT accounted for a large extent also for heterosis of rosette diameter at bolting and shoot biomass in Arabidopsis [29]. However, heterosis during the developmental vegetative stages before the transition to flowering were not influenced by FT [35]. In agreement with this, we did not find FT to correlate with LA at 17 DAS, a developmental stage at which none of the accessions or hybrids started to bolt. SY and NS were two other traits that were significantly correlated in parents, hybrids, and in MPH levels. Interestingly, no significant correlation was observed between SY and SW. This indicates that in Arabidopsis an enhanced total seed yield seems mostly determined by an elevated number of seeds produced by a plant and not by increased weight of individual seeds.

Eventually, none of the correlations between MPH levels of different traits were heterosis specific, showing that the genetic basis of heterosis is trait or trait-family specific and that heterosis for unrelated traits is not likely caused by the same genetic mechanism. Consequently, this implies that the ultimate hybrid, heterotic for multiple beneficial traits, may be hard to generate. This is supported by observations in rice hybrids, where an accumulation of numerous rare superior alleles leads to high heterotic values [42].

### Heterosis is independent of genetic distance between parents

In the past parental genetic distance has been proposed as a possible indicator to predict hybrid vigor (reviewed by [69]), fueled by the observation that interspecific hybrids generally show more heterosis than intraspecific hybrids [70]. Within species, correlations between genetic diversity and heterosis have been observed for several traits in for instance rice [71]. Moreover, whole-genome prediction was recently successfully applied to predict complex heterotic traits in maize [72]. In contrast, prediction of heterosis levels based on genetic distance between parents was highly trait dependent in Arabidopsis [22, 24, 27, 33] and maize [43]. Similarly, we could not detect any relationship between genetic distance of parental accessions and the level of heterosis exhibited by the hybrids for any of the traits under study. However, it should be noted that the accessions chosen in this study were part of a larger population that was specifically designed to contain the least possible population structure [44]. As a result the genetic distances between the common reference parents and all other accessions were maximised and rather similar. Preferably, the relationship between heterosis and genetic distance should be evaluated using a large number of hybrids derived from parents with a more diverse range of genetic distance values, similar to the set-up used by Riedelsheimer [72]. Nonetheless, the data obtained here suggest that in Arabidopsis the mechanisms underlying heterosis are not simply a function of genetic relatedness. In agreement with this, crosses between natural Arabidopsis accessions that were separated by similar geographic distances exhibited heterosis and outbreeding depression, respectively, the latter being a form of negative heterosis [33].

### GWA mapping of heterotic traits

The signals of association of single SNPs with heterosis in this study were often of low to moderate value. However, the associations for the traits under study in the parental lines were also not very strong, in contrast to the often single dominant peaks observed in GWA studies related to for instance (a)biotic stress adaptation [44, 73, 74] or flowering time [57]. In Maize GWA mapping of heterosis for several yield-related traits produced association signals of similar strength [72]. Together with the results obtained here this may imply that the genetic basis of yield related traits and thus heterosis for these traits are of a polygenic nature. Furthermore, only a limited number of lines were tested here, which severely limits detection power and conversely may increase significance levels of synthetic associations [75]. However, GWA analyses of heterotic traits in much larger populations of Arabidopsis and rice also suffered from low detection power [28, 40, 42]. In our experimental setup in general more SNPs with a dominant effect were found to be significantly associated with heterosis than SNPs with an additive effect for the parental trait means (S4 Table, S5 Table). However, it has been reported before that additive GWAs models may be underpowered [40].

Over the years three leading hypothesis have been established to explain the phenomenon of heterosis, namely dominance, overdominance and pseudo-overdominance. The identified SNPs/loci that were significantly associated with heterosis levels for various trait had predominantly a dominant effect (S5 Table) as had previously already been discussed by Seymour *et al*. [40]. However, the approach that was applied here does not allow easy discrimination between the established heterosis theories. Under the dominance hypothesis it is expected that heterosis is the result of complementation of various slightly deleterious alleles differentially present in both parents. As a consequence numerous loci with only small effect sizes are expected to contribute to heterosis (reviewed by [7]). Furthermore, under his theory heterosis is expected to increase with increasing genetic distance of the parental lines, simply because disadvantageous alleles in one parent have a higher chance of being complemented by the other parent when the parents are genetically more divers. In our study we predominantly found few associations with small effect and no correlation between heterosis levels and the genetic distance between the parental lines. Hence, the results as they were obtained here did do not fully support the dominance theory. In contrast, the overdominance hypothesis suggests a heterozygous advantage of specific alleles due to novel interactions between the parental alleles at a single heterozygous locus. Therefore only a limited number of loci with large effects are expected (reviewed by [7]). So far, only a few cases of heterosis caused by overdominance of a single allele have been described, in for instance Arabidopsis [30, 40], tomato [76], rice [41, 42] and humans [77]. However, in our study we did not identify large effect loci. Hence, the results described here did not favour one of the heterosis theories over the other. In addition, there is increasing evidence for a role of epigenetic regulation in heterosis [10, 12, 17], a mechanism not likely to be picked up by GWA mapping.

Nonetheless, interesting associations were found in our study that might incite further in-depth study. For example, two loci at the top of chromosome four that were associated with delayed FT and increased MSB in both hybrid-populations (S5 Table). The first locus co-localized with known flowering time genes including *FRI*. In Arabidopsis FRI is known to interact epistatically with FLC to suppress the transition to flowering [78]. Previously, at least 20 independent non-functional *FRI* haplotypes were detected, including the alleles in Col and L*er* [58]. However, both accessions still harbour functional *FLC* alleles [58]. Therefore we expected a delayed flowering phenotype in some of our hybrids due to possible complementation of the non-functional *FRI* alleles of both common reference parents by functional *FRI* alleles present in some of the parental lines. Indeed, one of the associations with FT heterosis was found to be co-localizing with *FRI*. However, SNPs within *FRI* itself were not significantly associated in our study, in line with previous observations [44]. The additional locus at chromosome four associated with FT heterosis did not harbour any known flowering time genes and remains an interesting candidate for further study.

### Heterosis remains an elusive trait

In conclusion, additional studies are required to elucidate the molecular mechanisms underlying heterosis. The results shown here indicate that the genetic mechanisms underlying heterosis are highly trait specific and depend greatly on the genetic background. This indicates that different mechanisms may be in play and that the number of heterotic loci is nearly infinite, which makes heterosis a highly elusive trait. Nonetheless, given the agro-economic importance of this trait, additional study of heterosis remains extremely valuable. However, it is recommended that efforts should focus on individual traits in environments where heterosis can make a difference.

## Supporting information

S1 Fig Frequency distribution of trait values for all lines in population 1 and 2. Classes to which the common reference parents Col-0 coloured seed in population 1 (green) and L*er*-0^*ms/ms*^ in population 2 (red) belong to are represented by hatched lines. Abbreviations: LA, projected leaf area in cm^2^ at 17 days after sowing; FT, flowering time in days after sowing of opening of first flower; HT, height of the inflorescence in cm; MSB, main stem branching in number of branches originated on the main stem; RB, rosette branching in number of branches originated from the base of the rosette; SY, seed yield in grams of seeds produced per plant; NS, number of seeds produced per plant; SZ, seed size; SW, 1000 seed weight in estimated grams of 1000 seeds.
(PDF)

S2 Fig Ranges of mid and best parent heterosis for height in both hybrid populations. Shown are MPH and BPH ranges for HT for Col hybrids (depicted by light and dark green bars respectively) and L*er* hybrids (depicted by red and purple bars respectively).
(PDF)

S3 Fig Growth curves of hybrids and parental lines of population 2. Depicted are the average projected leaf areas in mm^2^ of approx. 10 replicates of individual hybrid genotypes (represented by dark gray lines) and parental genotypes (represented by light gray lines) in population 2 over time (hours after sowing). Red dots indicate when actual measurements were taken. The red line represents the growth of the common reference line L*er msms*.
(PDF)

S4 Fig Differences in plant establishment between L*er*-hybrids and associated parental lines. Depicted are the population mean of hybrids and parental lines of the percentage of visible plants using automatic imaging on five different timepoints (days after sowing). Error bars represent standard errors. Asterisks indicate significant differences between hybrid population and parental population (Student’s t-test, α = 0.05).
(PDF)

S5 Fig Heterosis for projected leaf area in the L*er* hybrid population. (**A**) MPH ranges for LA at 17 DAS before correction (light bars) and at 8 days after correction taking 2mm^2^ as t_0_ (dark bars). (**B**) MPH for LA over time, after correction. Different colors indicate MPH levels of different hybrid lines.
(PDF)

S6 Fig Genome wide association profiles for all traits for parental lines and MPH_ABS_ values of both common reference populations. Manhattan plots representing the associations between SNP markers and the traits under study. Individual graphs are named in a tripartite manner. The first part indicates the studied trait, namely LA, FT, HT, MSB, RB, SY, SW, NS, and SZ (trait abbreviations as listed in the legend of Table 1). The second part refers to mean of parental lines (MeanP) or absolute MPH levels (MPH_abs). The last part refers to the population in which the trait was studied, namely population 1, the Col-hybrid population and its associated parental lines (Col), and population 2, the L*er*-hybrid population and its associated parental lines (L*er*). X-axis displays the basepair position along the Arabidopsis genome, with red and blue indicating the 5 different chromosomes. Y-axis displays -log_10_(*P*-value). Dotted line represent the significance threshold, which was set at -log_10_(*P*-value) > 4.
(PDF)

S1 Table *Arabidopsis thaliana* accessions used in this study. (XLSX)

S2 Table Comparison of different pollination strategies for all traits. Data shown are means of 4–30 plants ±SD for 9 different traits. LA, projected leaf area 17 days after sowing in cm^2^; FT, flowering time in days after sowing of opening of first flower; HT, height of the inflorescence in cm; MSB, main stem branching in number of side branches on the main inflorescence; RB, rosette branching in number of branches originating from the rosette in addition to the main stem; SY, seed yield in gram of seeds per plant; NS, number of seeds per plant; SZ, seed size; SW, 1000 seed weight; SD, standard deviation; Self, self-pollination; Manual, manual pollination of five to six non-emasculated young flowers on the main stem while retaining axillary flower meristems intact; Sig., different letters indicate significant differences between the lines (Student’s t-test, P < 0.05).
*^1^Plants were pooled prior to measurement of SY, NS, SZ, SW in population 1 and NS, SZ, SW in population 2.
*^2^Measurements of HT, MSB, RB, and seed related traits were only conducted on those plants derived from the L*er Msms* x L*er Msms* cross that were fertile.
(XLSX)

S3 Table Correlation between heterosis levels and genetic distances of parental lines. Spearman's rank correlations (*r*_*s*_) between mid parent heterosis levels (MPH) and genetic distances between parental lines (GD) were calculated, using the Arabidopsis Kinship matrix [50] as a proxy for GD. Numbers in bold indicate significant correlations (p<0.05). Abbreviations: LA, projected leaf area 17 days after sowing in cm^2^; FT, flowering time in days after sowing of opening of first flower; HT, height of the inflorescence in cm; MSB, main stem branching in number of side branches on the main inflorescence; RB, rosette branching in number of branches originating from the rosette in addition to the main stem; SY, seed yield in gram of seeds per plant; NS, number of seeds per plant; SZ, seed size; SW, 1000 seed weight; GD, genetic distance; MPH, mid parent heterosis.
(XLSX)

S4 Table Number of significant SNPs for various traits identified by genome wide association analysis for MPH_ABS_ and trait means of parental lines. Given are the number of SNPs for each trait that are significantly associated using–log_10_(*P*-value) > 4 as cut-off (number before slash), or at p<0.05 after *P*-value correction for multiple testing according to the Benjamini and Hochbergs's FDR-controlling procedure (number after slash). LA, projected leaf area 17 days after sowing; FT, flowering time in days after sowing of opening of first flower; HT, height of the inflorescence in cm; MSB, main stem branching in number of side branches on the main inflorescence; RB, rosette branching in number of branches originating from the rosette in addition to the main stem; SY, seed yield; SW, 1000 seed weight; SZ, seed size and NS, number of seeds produced per plant.
(XLSX)

S5 Table Significant SNPs for various traits identified by genome wide association analysis for parental lines and MPH_ABS_ levels. Given are–log_10_(*P*-value) and estimated effect sizes (*in italics*) of all SNPs that were significantly associated with any of the traits under study in population 1 (Col) or population 2 (L*er*) using a–log_10_(*P*-value)> 4 as cut-off. Effect sizes estimated for MPH_ABS_ reflect dominant effect sizes (d), while effect sizes estimated for the parental means reflect additive effect sizes (2a). Significant SNPs are highlighted according to their reported–log_10_(*P*-value) of respectively 4–5 (in yellow), 5–6 (in orange), and >6 (in red). Genes in the vicinity of the selected SNPs are given based on their position. However, it should be noted that these genes may not necessarily be the causal genes. -log_10_(*P*-values) highlighted in bold indicate SNPs that still showed a significant association after *P*-value correction for multiple testing according to the Benjamini and Hochbergs's FDR-controlling procedure at p<0.05 (see S7 Table for FDR corrected *P*-values). Used abbreviations: LA, projected leaf area 17 days after sowing; FT, flowering time in days after sowing of opening of first flower; HT, height of the inflorescence in cm; MSB, main stem branching in number of side branches on the main inflorescence; RB, rosette branching in number of branches originating from the rosette in addition to the main stem; SY, seed yield; SW, 1000 seed weight; SZ, seed size and NS, number of seeds produced per plant.
(XLSX)

S6 Table Raw data file for all traits under study in individual parental lines and hybrids in both common reference hybrid populations. First column indicates genotype id, which is named after parental accessions. Naming of traits under study is as described in legend S6 Fig. Where possible, trait measurements are given for individual plant replicas, however, for conveniences the order of plant replicas was not kept intact between traits.
(XLSX)

S7 Table FDR corrected *P*-values. Given are *P*-values after correction for multiple testing according to the Benjamini and Hochbergs's FDR-controlling procedure for all SNPs in the GWA analysis for the traits under study in population 1 (Col) or population 2 (L*er*). Genes in the vicinity of the selected SNPs are given based on their position. Used abbreviations: Chrom., chromosome; SE, standard error; LA, projected leaf area 17 days after sowing; FT, flowering time in days after sowing of opening of first flower; HT, height of the inflorescence in cm; MSB, main stem branching in number of side branches on the main inflorescence; RB, rosette branching in number of branches originating from the rosette in addition to the main stem; SY, seed yield; SW, 1000 seed weight; SZ, seed size and NS, number of seeds produced per plant.
(XLSX)
