# Supplementary material for: Assessment of heterosis in two Arabidopsis thaliana common-reference mapping populations
Source: PLoS One. 2018 Oct 12;13(10):e0205564. doi: 10.1371/journal.pone.0205564 (PMC6185836; doi:10.1371/journal.pone.0205564)
Supplement: S1 Fig — Classes to which the common reference parents Col-0 coloured seed in population 1 (green) and Ler-0ms/ms in population 2 (red) belong to are represented by hatched lines. Abbreviations: LA, projected leaf area in cm2 at 17 days after sowing; FT, flowering time in days after sowing of opening of first flower; HT, height of the inflorescence in cm; MSB, main stem branching in number of branches originated on the main stem; RB, rosette branching in number of branches originated from the base of the rosette; SY, seed yield in grams of seeds produced per plant; NS, number of seeds produced per plant; SZ, seed size; SW, 1000 seed weight in estimated grams of 1000 seeds. (PDF) [file pone.0205564.s001.pdf]

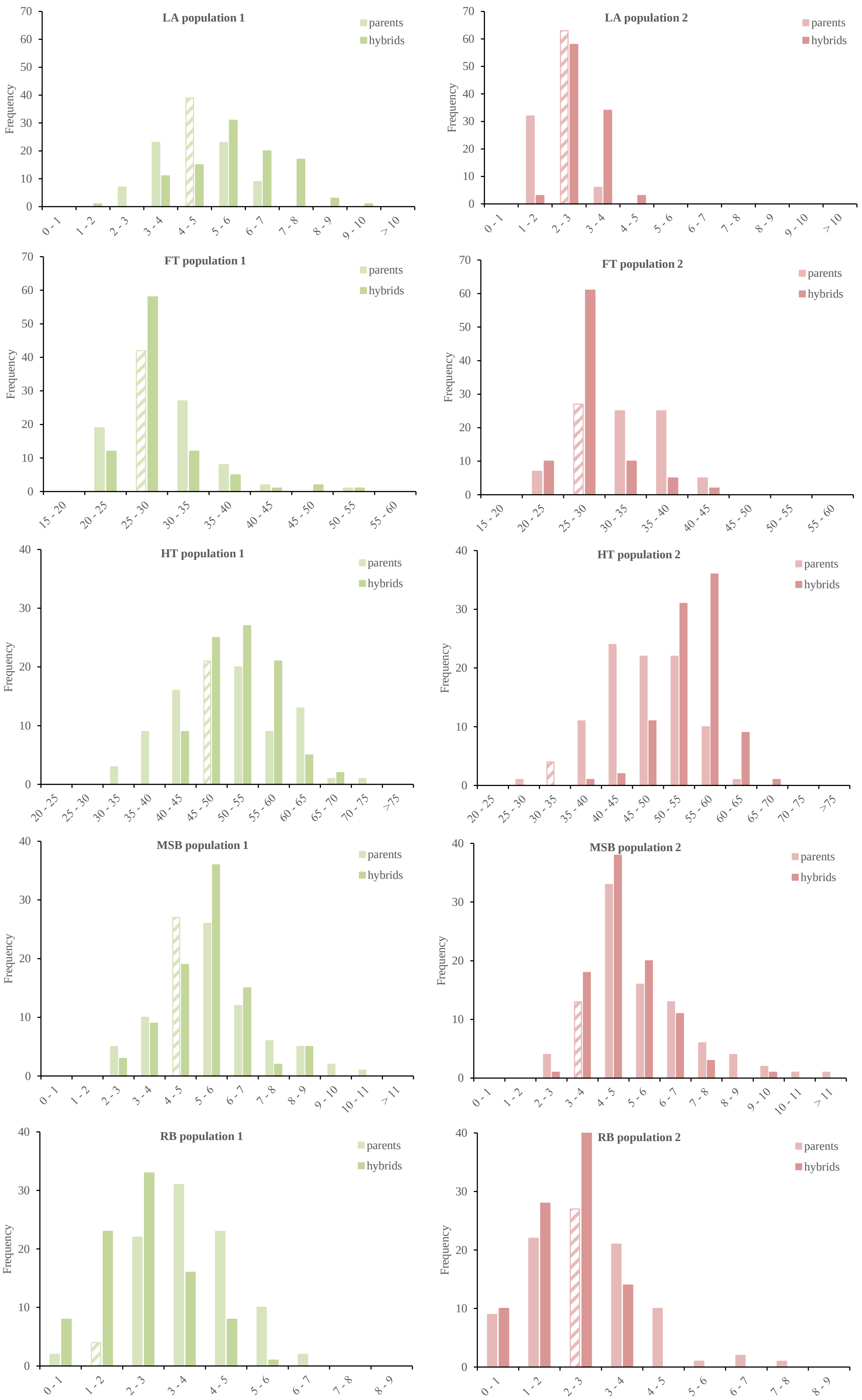

LA population 1
parents
hybrids
Frequency
0 - 1
1 - 2
2 - 3
3 - 4
4 - 5
5 - 6
6 - 7
7 - 8
8 - 9
9 - 10
> 10
LA population 2
FT population 1
15 - 20
20 - 25
25 - 30
30 - 35
35 - 40
40 - 45
45 - 50
50 - 55
55 - 60
FT population 2
HT population 1
20 - 25
25 - 30
30 - 35
35 - 40
40 - 45
45 - 50
50 - 55
55 - 60
60 - 65
65 - 70
70 - 75
>75
HT population 2
MSB population 1
10 - 11
> 11
MSB population 2
RB population 1
RB population 2

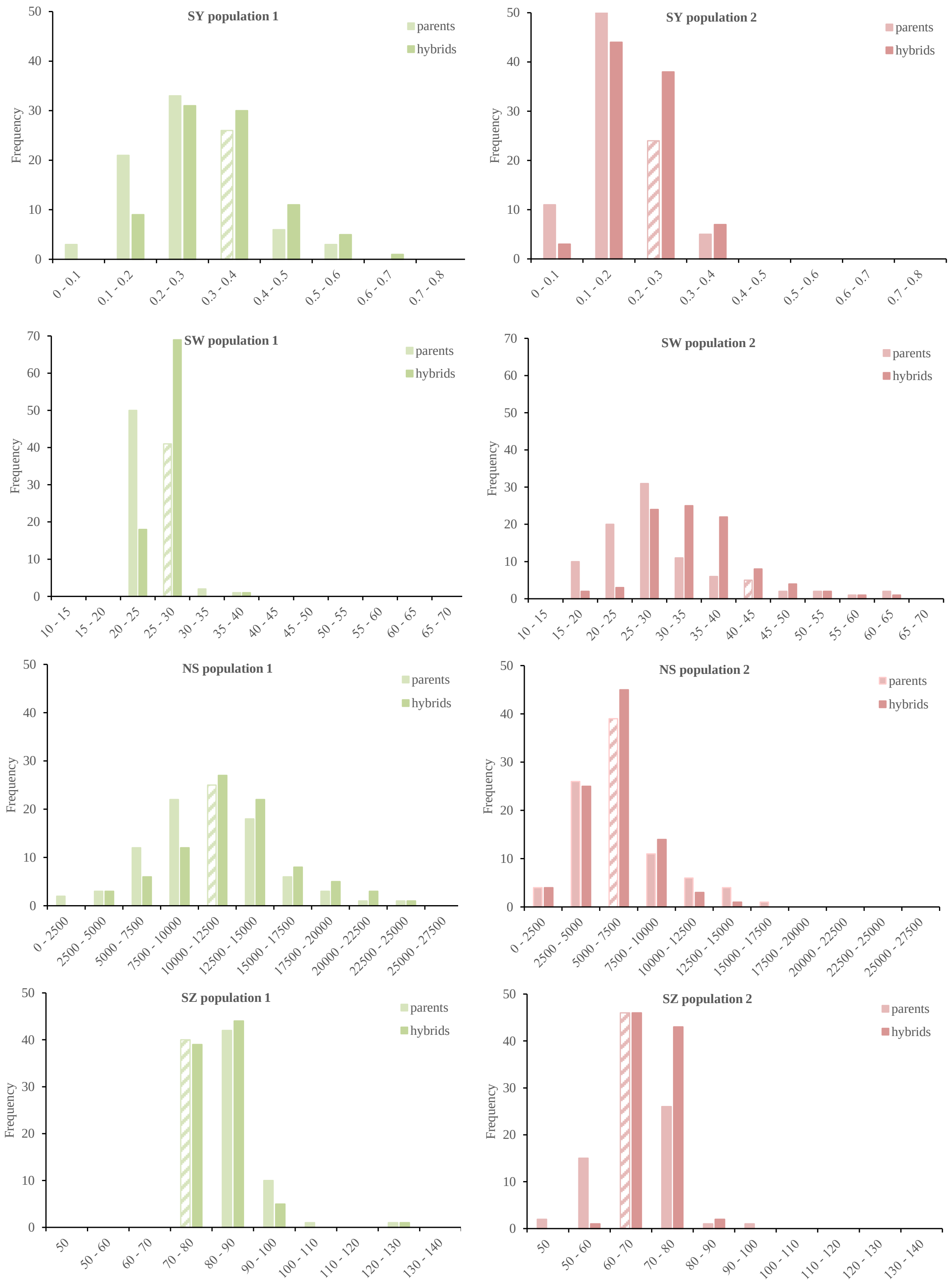

S1 Fig: Frequency distribution of trait values for all lines in population 1 and 2. Classes to which the common reference parents Col-0 coloured seed in population 1 (green) and L*er*-0 *msms* in population 2 (red) belong to are hatched. Abbreviations: LA, projected leaf area in cm^2 at 17 days after sowing; FT, flowering time in days after sowing of opening of first flower; HT, height of the inflorescence in cm; MSB, main stem branching in number of branches originated on the main stem; RB, rosette branching in number of branches originated from the base of the rosette; SY, seed yield in grams of seeds produced per plant; NS, number of seeds produced per plant; SW, 1000 seed weight in estimated grams of 1000 seeds.
