# Supplementary material for: Assessment of heterosis in two Arabidopsis thaliana common-reference mapping populations
Source: PLoS One. 2018 Oct 12;13(10):e0205564. doi: 10.1371/journal.pone.0205564 (PMC6185836; doi:10.1371/journal.pone.0205564)
Supplement: S2 Fig — Shown are MPH and BPH ranges for HT for Col hybrids (depicted by light and dark green bars respectively) and Ler hybrids (depicted by red and purple bars respectively). (PDF) [file pone.0205564.s002.pdf]

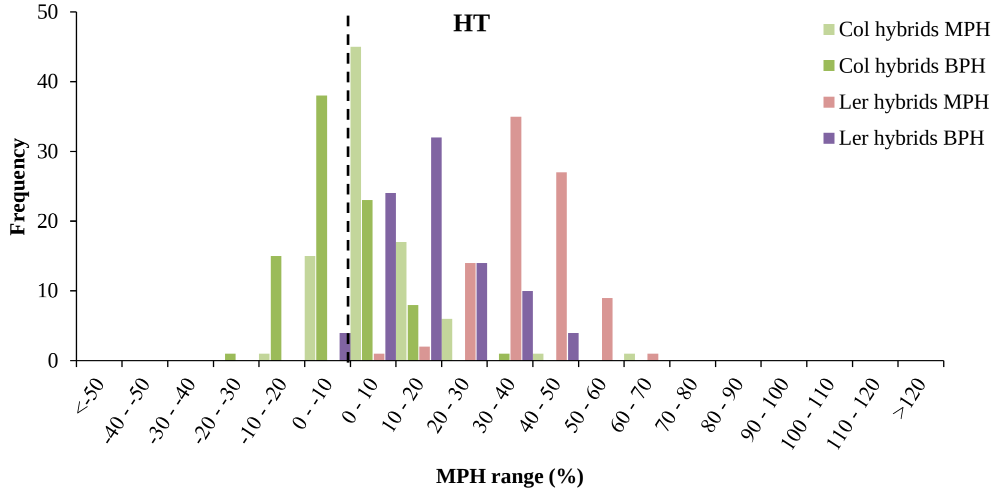

S2 Fig: Ranges of mid and best parent heterosis for height in both hybrid populations. Shown are MPH and BPH ranges for HT for Col hybrids (depicted by light and dark green bars respectively) and L*er* hybrids (depicted by red and purple bars respectively)
