# Supplementary material for: Assessment of heterosis in two Arabidopsis thaliana common-reference mapping populations
Source: PLoS One. 2018 Oct 12;13(10):e0205564. doi: 10.1371/journal.pone.0205564 (PMC6185836; doi:10.1371/journal.pone.0205564)
Supplement: S3 Fig — Depicted are the average projected leaf areas in mm2 of approx. 10 replicates of individual hybrid genotypes (represented by dark gray lines) and parental genotypes (represented by light gray lines) in population 2 over time (hours after sowing). Red dots indicate when actual measurements were taken. The red line represents the growth of the common reference line Ler msms. (PDF) [file pone.0205564.s003.pdf]

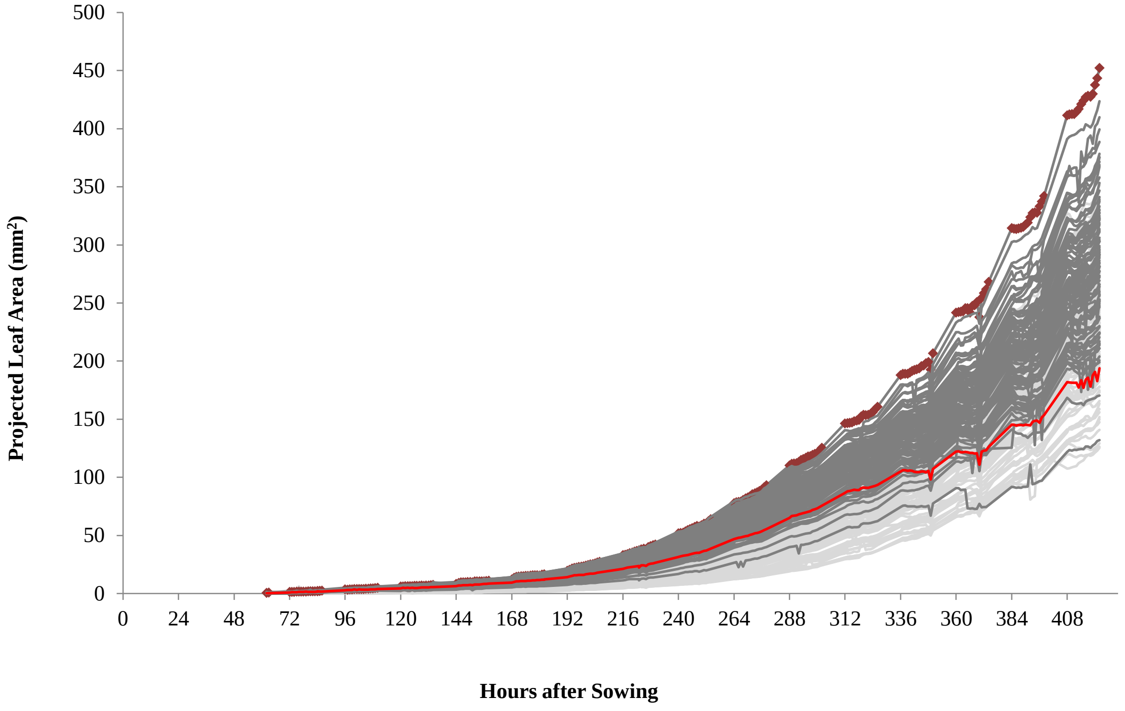

S3 Fig: Growth curves of hybrids and parental lines of population 2. Depicted are the average projected leaf areas in mm^2 of approx. 10 replicates of individual hybrid genotypes (represented by dark gray lines) and parental genotypes (represented by light gray lines) in population 2 over time (hours after sowing). Red dots indicate when actual measurements were taken. The red line represents the growth of the common reference line L*er* msms.
