# Supplementary material for: Assessment of heterosis in two Arabidopsis thaliana common-reference mapping populations
Source: PLoS One. 2018 Oct 12;13(10):e0205564. doi: 10.1371/journal.pone.0205564 (PMC6185836; doi:10.1371/journal.pone.0205564)
Supplement: S4 Fig — Depicted are the population mean of hybrids and parental lines of the percentage of visible plants using automatic imaging on five different timepoints (days after sowing). Error bars represent standard errors. Asterisks indicate significant differences between hybrid population and parental population (Student’s t-test, α = 0.05). (PDF) [file pone.0205564.s004.pdf]

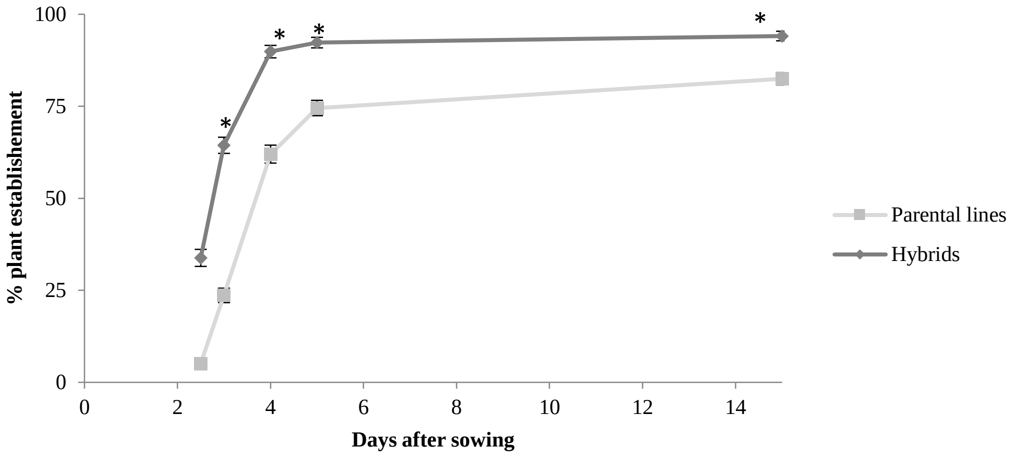

S4 Fig: Differences in plant establishment between Ler-hybrids and associated parental lines. Depicted are the population mean of hybrids and parental lines of the percentage of visible plants using automatic imaging on five different timepoints (days after sowing). Error bars represent standard errors. Asterisks indicate significant differences between hybrid population and parental population (Student’s t-test, $\alpha = 0.05$).
