# Supplementary material for: Assessment of heterosis in two Arabidopsis thaliana common-reference mapping populations
Source: PLoS One. 2018 Oct 12;13(10):e0205564. doi: 10.1371/journal.pone.0205564 (PMC6185836; doi:10.1371/journal.pone.0205564)
Supplement: S5 Fig — (A) MPH ranges for LA at 17 DAS before correction (light bars) and at 8 days after correction taking 2mm2 as t0 (dark bars). (B) MPH for LA over time, after correction. Different colors indicate MPH levels of different hybrid lines. (PDF) [file pone.0205564.s005.pdf]

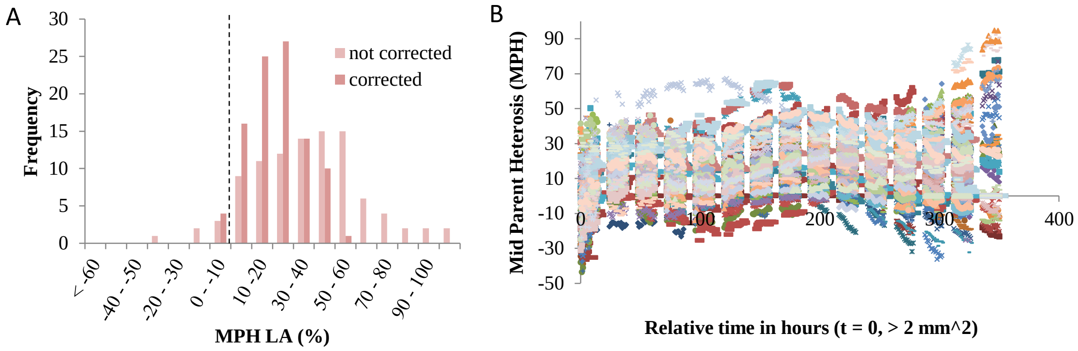

S5 Fig: Heterosis for projected leaf area in the L*er* hybrid population. (**A**) MPH ranges for LA at 17 DAS before correction (light bars) and at 8 days after correction taking 2mm^2 as t_0 (dark bars). (**B**) MPH for LA over time, after correction. Different colors indicate MPH levels of different hybrid lines.
