# Supplementary material for: Assessment of heterosis in two Arabidopsis thaliana common-reference mapping populations
Source: PLoS One. 2018 Oct 12;13(10):e0205564. doi: 10.1371/journal.pone.0205564 (PMC6185836; doi:10.1371/journal.pone.0205564)
Supplement: S6 Fig — Manhattan plots representing the associations between SNP markers and the traits under study. Individual graphs are named in a tripartite manner. The first part indicates the studied trait, namely LA, FT, HT, MSB, RB, SY, SW, NS, and SZ (trait abbreviations as listed in the legend of Table 1). The second part refers to mean of parental lines (MeanP) or absolute MPH levels (MPH_abs). The last part refers to the population in which the trait was studied, namely population 1, the Col-hybrid population and its associated parental lines (Col), and population 2, the Ler-hybrid population and its associated parental lines (Ler). X-axis displays the basepair position along the Arabidopsis genome, with red and blue indicating the 5 different chromosomes. Y-axis displays -log10(P-value). Dotted line represent the significance threshold, which was set at -log10(P-value) > 4. (PDF) [file pone.0205564.s006.pdf]

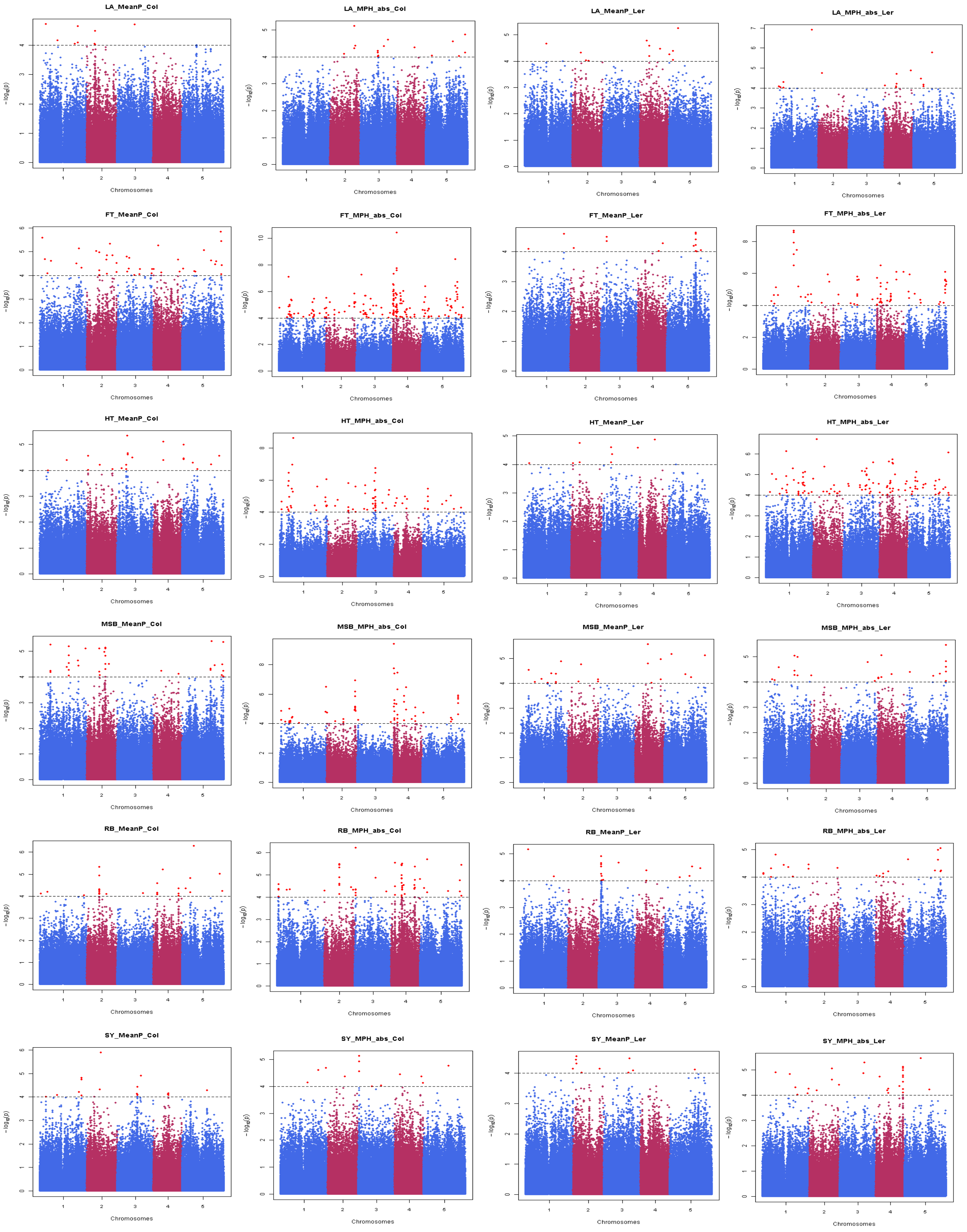
LA_MeanP_Col
LA_MPH_abs_Col
LA_MeanP_Ler
LA_MPH_abs_Ler
FT_MeanP_Col
FT_MPH_abs_Col
FT_MeanP_Ler
FT_MPH_abs_Ler
HT_MeanP_Col
HT_MPH_abs_Col
HT_MeanP_Ler
HT_MPH_abs_Ler
MSB_MeanP_Col
MSB_MPH_abs_Col
MSB_MeanP_Ler
MSB_MPH_abs_Ler
RB_MeanP_Col
RB_MPH_abs_Col
RB_MeanP_Ler
RB_MPH_abs_Ler
SY_MeanP_Col
SY_MPH_abs_Col
SY_MeanP_Ler
SY_MPH_abs_Ler
Chromosomes
$-\log_{10}(p)$

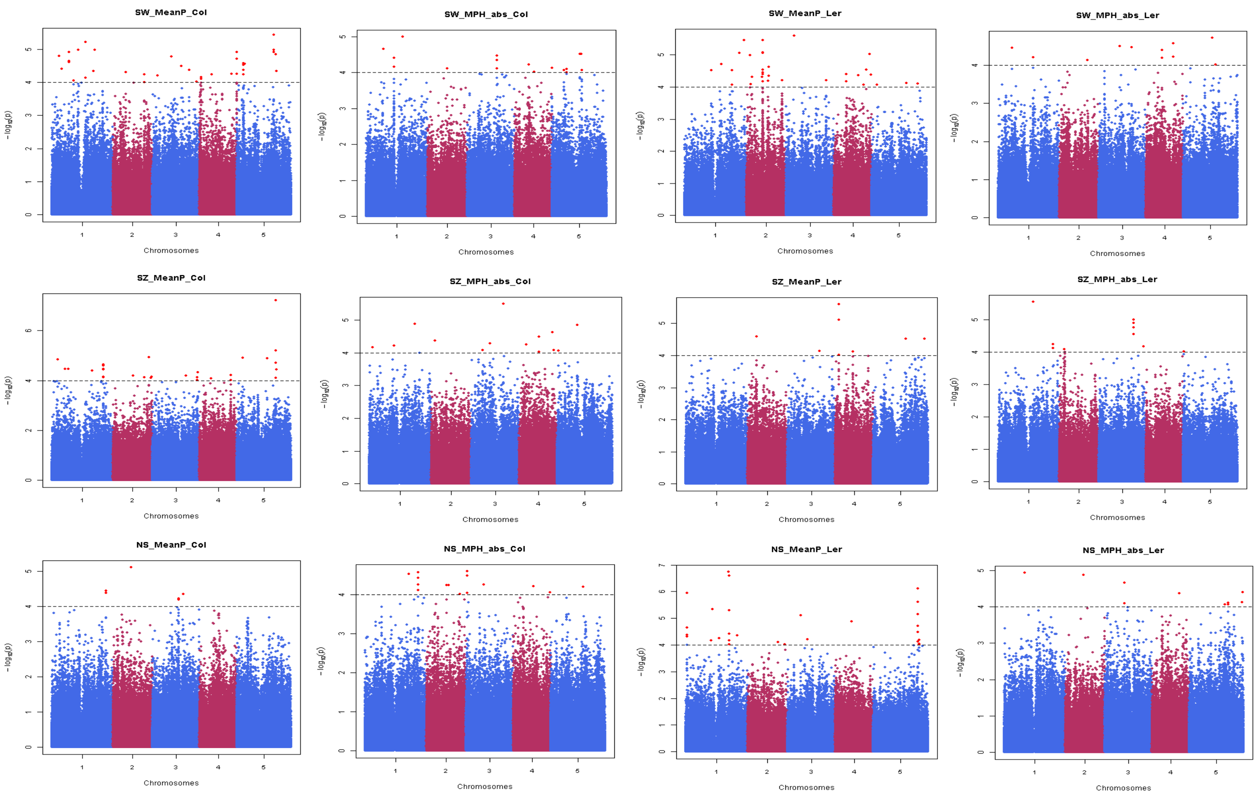

S6 Fig: Genome wide association profiles for all traits for parental lines and MPH_{ABS} values of both common reference populations. Manhattan plots representing the associations between SNP markers and the traits under study. Individual graphs are named in a tripartite manner. The first part indicates the studied trait, namely LA, FT, HT, MSB, RB, SY, SW, NS, and SZ (trait abbreviations as listed in the legend of table 1). The second part refers to mean of parental lines (MeanP) or absolute MPH levels (MPH_abs). The last part refers to the population in which the trait was studied, namely population 1, the Col-hybrid population and its associated parental lines (Col), and population 2, the L*er*-hybrid population and its associated parental lines (Ler). X-axis displays the basepair position along the Arabidopsis genome, with red and blue indicating the 5 different chromosomes. Y-axis displays -$\log_{10}$(*P*-value). Dotted line represent the significance threshold, which was set at -$\log_{10}$(*P*-value) > 4.
